# Enhanced Mutant Compensates for Defects in Rhodopsin Phosphorylation in the Presence of Endogenous Arrestin-1

**DOI:** 10.3389/fnmol.2018.00203

**Published:** 2018-06-18

**Authors:** Srimal Samaranayake, Xiufeng Song, Sergey A. Vishnivetskiy, Jeannie Chen, Eugenia V. Gurevich, Vsevolod V. Gurevich

**Affiliations:** ^1^Department of Pharmacology, Vanderbilt University, Nashville, TN, United States; ^2^Zilkha Neurogenetic Institute, Keck School of Medicine, University of Southern California, Los Angeles, CA, United States

**Keywords:** rod, photoreceptor, arrestin-1, rhodopsin, phosphorylation, compensation

## Abstract

We determined the effects of different expression levels of arrestin-1-3A mutant with enhanced binding to light-activated rhodopsin that is independent of phosphorylation. To this end, transgenic mice that express mutant rhodopsin with zero, one, or two phosphorylation sites, instead of six in the WT mouse rhodopsin, and normal complement of WT arrestin-1, were bred with mice expressing enhanced phosphorylation-independent arrestin-1-3A mutant. The resulting lines were characterized by retinal histology (thickness of the outer nuclear layer, reflecting the number of rod photoreceptors, and the length of the outer segments, which reflects rod health), as well as single- and double-flash ERG to determine the functionality of rods and the rate of photoresponse recovery. The effect of co-expression of enhanced arrestin-1-3A mutant with WT arrestin-1 in these lines depended on its level: higher (240% of WT) expression reduced the thickness of ONL and the length of OS, whereas lower (50% of WT) expression was harmless in the retinas expressing rhodopsin with zero or one phosphorylation site, and improved photoreceptor morphology in animals expressing rhodopsin with two phosphorylation sites. Neither expression level increased the amplitude of the a- and b-wave of the photoresponse in any of the lines. However, high expression of enhanced arrestin-1-3A mutant facilitated photoresponse recovery 2-3-fold, whereas lower level was ineffective. Thus, in the presence of normal complement of WT arrestin-1 only supra-physiological expression of enhanced mutant is sufficient to compensate for the defects of rhodopsin phosphorylation.

## Introduction

Rods are dim light receptors in the retina with remarkable single-photon sensitivity (Baylor et al., 1979). Rod phototransduction is initiated when the photon activates rhodopsin molecule by isomerization of 11-*cis*-retinal covalently attached to it into all-*trans*-retinal (Baylor et al., 1979). Light-activated rhodopsin then sequentially activates a number of transducin molecules (Arshavsky and Burns, 2014). Active transducin Gα-GTP in its turn activates cGMP phosphodiesterase, which reduces cGMP concentration in the rod outer segment by converting it to GMP, thereby causing the closure of cGMP-gated channels (Gross et al., 2015). The signaling is terminated by a two-step mechanism common to most G protein-coupled receptors (GPCRs): phosphorylation by rhodopsin kinase [systematic name GRK1 (Gurevich et al., 2012)], whereupon visual arrestin-1^1^ specifically binds active phosphorylated rhodopsin precluding further transducin activation (Burns and Pugh, 2010) via direct competition (Wilden, 1995; Krupnick et al., 1997). The breakdown of this inactivation mechanism due to lack of rhodopsin kinase, arestin-1, or the absence of phosphorylation sites on rhodopsin results in Oguchi disease (a form of night blindness) or retinal degeneration in humans (Apfelstedt-Sylla et al., 1993; Fuchs et al., 1995; Cideciyan et al., 1998), as well as the loss of rod function and retinal degeneration in mice (Chen et al., 1995; Xu et al., 1997; Chen C.K. et al., 1999; Chen J. et al., 1999). Excessive signaling due to gain-of-function mutations in non-visual GPCRs also causes various disorders (Schöneberg et al., 2004; Stoy and Gurevich, 2015). In contrast to recessive loss-of-function mutations, gain-of-function mutations in GPCRs are dominant, i.e., a perfectly normal protein encoded by the second allele does not prevent excessive signaling of the mutant. Our structure-function studies of visual arrestin-1 yielded a number of “enhanced” mutants that bind all functional forms of rhodopsin more readily and interact with fairly high affinity with active unphosphorylated rhodopsin (Gurevich and Benovic, 1993, 1995; Gurevich, 1998; Vishnivetskiy et al., 1999, 2013). Most importantly, enhanced arrestin-1 can quench the signaling by unphosphorylated rhodopsin (Gray-Keller et al., 1997). We found that transgenic expression of an enhanced arrestin-1-3A mutant can partially compensate for the defects in rhodopsin phosphorylation, improving the morphology and function of rods lacking rhodopsin kinase and speeding up photoresponse recovery (Song et al., 2009). However, encouraging, this study had two limitations: compensational potential of the mutant was tested only in one model of defective rhodopsin phosphorylation, rhodopsin kinase knockout mice, and only in the absence of endogenous wild type (WT) arrestin-1 (Song et al., 2009). In this situation the lower (about 50% of WT) expression of enhanced mutant turned out to be more beneficial than the higher (∼240% of WT) level (Song et al., 2009), likely because high mutant expression caused fairly rapid light-independent retinal degeneration (Song et al., 2013). Lower expression level of the arrestin-1-3A mutant also caused retinal degeneration in arrestin knockout mice, albeit much slower, but co-expression of WT arrestin-1 with the mutant expressed at ∼50% of WT arrestin level appeared to protect rods (Song et al., 2013). Here we tested the ability of the same enhanced mutant expressed at both levels to compensate for defects of phosphorylation in mice expressing rhodopsin with limited number or complete absence of phosphorylation sites, which cannot be quenched by a normal mechanism (Mendez et al., 2000a), in the presence of normal level of endogenous WT arrestin-1, which mimics the situation in human patients.

## Materials and Methods

### Ethics Statement

Animal research was conducted in compliance with the NIH Guide for the Care and Use of Laboratory Animals and approved by the Vanderbilt University Institutional Animal Care and Use Committee/Office of Animal Welfare Assurance (protocol ID M/15/090).

### Generation of Transgenic Mice Expressing Arrestin-1 at Different Levels

The generation of transgenic animals expressing rhodopsin mutants with 0, 1, or 2 phosphorylation sites on the background of rhodopsin knockout (Mendez et al., 2000a,b), as well as enhanced arrestin-1-3A mutant at different levels under the pRho4-1 rhodopsin promoter (Nair et al., 2005; Chan et al., 2007; Song et al., 2009) on C57bl background has been described previously. As it has been established that reduced (Lem et al., 1999), as well as increased (Li et al., 1996; Mendez et al., 2000b) relative to WT levels of rhodopsin expression are detrimental for rods, we used previously characterized lines expressing rhodopsin mutants at the levels close to that of WT rhodopsin in mouse rods (Mendez et al., 2000a,b). The lines expressing rhodopsin mutants on the background of rhodopsin knockout used here were shown to maintain normal retinal morphology up to 2 months of age, except phosphorylation-deficient CSM line that exhibited slow retinal degeneration (Mendez et al., 2000a,b). After 3–4 generations all lines were backcrossed to WT C57bl mice (Charles River, Wilmington, MA, United States) to assure unaltered genetic background. The arrestin-1-3A mutant used in this study has triple alanine substitution in the arrestin C-terminus that detaches it from the body of the molecule, thereby facilitating rhodopsin binding, with the greatest effect on the binding to light-activated unphosphorylated rhodopsin in bovine (Gurevich, 1998) and mouse (Song et al., 2009; Vishnivetskiy et al., 2013) arrestin-1.

### Histological Analysis

Mice were maintained in the animal facility under ambient illumination on a 12 hr light/dark cycle. Histological analysis was performed, as described (Song et al., 2011). Briefly, at the age of 5–6 weeks, mice were sacrificed by overdose of isoflurane, the eyes were enucleated and fixed in 4% paraformaldehyde (Sigma-Aldrich, St Louis, MO, United States) at 4°C overnight, cryoprotected with 30% sucrose (Sigma-Aldrich) in phosphate-buffered saline (PBS) (Sigma-Aldrich) for 6 h, and frozen at -80°C. Sections (30 μm) were cut using Cryostat CM3050S (Leica, Germany) and mounted on Vectabond (Vector Laboratories, Burlingame, CA, United States) coated slides. The sections were rehydrated for 40 min in 0.1M PBS, pH 7.2, then incubated for 10 min in PBS with 0.1% Triton X-100, washed twice for 5 min with PBS, and stained with NeuroTrace 500/525 green fluorescent Nissl stain (Invitrogen, Carlsbad, CA, United States) in PBS (dilution 1:100) for 20 min. The sections were then washed with PBS with 0.1% Triton X-100 for 10 min, rinsed twice with PBS (5 min each), and kept overnight at 4°C in PBS. Mounted sections were analyzed by confocal microscopy (LSM510; Zeiss, Oberkochen, Germany). The outer nuclear layer (ONL) was visualized by fluorescence in FITC (green) channel, outer segments (OS) were viewed with DIC (differential interference contrast). Two-channel images were acquired for quantitative analysis. Nissl stains nucleic acids, labeling DNA-rich nuclei in the outer nuclear layer, and yielding more diffuse staining of RNA-rich inner segments. It also helps to identify the outer segments that are essentially free of nucleic acids and therefore contain no Nissl stain, but are clearly visible in DIC image.

The length of the OS and the thickness of ONL were measured using NIS-Elements software (Nikon Metrology Inc, Brighton, MI, United States). The retinas of three mice (three sections per mouse) for each genotype were used. Each side of the section was divided into three segments according to the distance from optical nerve: central, medium, and peripheral in both inferior and superior hemispheres, so that the whole length of the section was divided into six segments. The measurements were performed at five points spaced an equal distances within each segment. Average values of each parameter for individual animals was used to calculate the mean and SD for each genotype.

### Electroretinography (ERG)

Electroretinograms were recorded from 5 to 6 week old mice reared in 12/12 light-dark cycle (90 ± 10 lux in the cage during light period). Mice were dark-adapted overnight (>12 h), as described (Song et al., 2009, 2011). Under dim red light animals were anesthetized by an IP injection of (shown as μg/g body weight) 15–20 ketamine, 6–8 xylazine, 600–800 urethane in PBS. The pupils were dilated with 1% tropicamide in PBS throughout the experiment. An eye electrode made with a coiled 0.2 mm platinum wire (Lyubarsky et al., 2002) was placed on the cornea, a needle reference electrode was placed in the cheek, and ground needle electrode in the tail (Lyubarsky and Pugh, 1996; Lyubarsky et al., 2002, 2004). ERG data were collected using the UTAS E-3000 system (LKC Technologies, Inc, Gaithersburg, MD, United States) connected to a Ganzfeld chamber that produced brief (20 μs to 1 ms) full field flash stimuli. Calibrated by the manufacturer light intensities were computer controlled. As mice are temperature-sensitive, animals were kept on a heating pad connected to a temperature control unit to maintain the temperature at 37–38°C throughout the experiment.

#### Single-Flash Protocol

A series of stimuli from 0.00025 to 138 cd × s/m^2^ (or -3.6 to 2.15 log cd × s/m^2^, covering the range of 5.75 log units) in steps of 0.2 log units was presented in an ascending order. Sufficient time interval between flashes (from 20 s for low intensity flashes up to 3 min for the highest intensity) was allowed for dark adaptation. To increase precision, the responses to dim flashes were recorded two-three times. The a-wave amplitude was measured from baseline to the bottom of the a-wave trough, and the b-wave amplitude was measured from the bottom of the a-wave trough to the b-wave peak.

#### Double-Flash Protocol

The double flash protocol was used to analyze the kinetics of photoresponse recovery (Pepperberg et al., 1992, 1997; Lyubarsky and Pugh, 1996; Hetling and Pepperberg, 1999; Lyubarsky et al., 2002; Song et al., 2009, 2011). A test flash [-0.4 log cd × s/m^2^, corresponding to ∼400 photoisomerizations per rod (Lyubarsky et al., 2004)] was delivered to suppress the circulating current of the rod photoreceptors. The recovery was monitored by delivering a second (probe) flash [0.65 log cd × s/m^2^, corresponding to ∼4,500 photoisomerizations per rod (Lyubarsky et al., 2004)] at indicated time intervals after the test flash. Sufficient time for dark adaptation was allowed between trials, as shown by the reproducibility of the response to the test flash. Time-to-peak (implicit time) of the a-wave after the probe flash was not significantly different in all genotypes. This finding and the shape of the a-wave shows that the intrusion of b-wave and oscillation potentials (Pepperberg et al., 1997; Hetling and Pepperberg, 1999) did not differentially affect different genotypes. The response to a probe flash recorded without a preceding test flash was used to normalize the responses to probe flashes following a test flash (to calculate 1-a/a_max_, where a is the response to the probe flash after each time interval, whereas a_max_ is the control response to the probe flash without preceding test flash). The normalized amplitude of the probe flash a-wave was plotted as a function of time between the two flashes. The data points were fitted to polynomial non-linear regression using GraphPad Prism (Version 4.0) (*R*^2^ > 0.95 was considered as a criterion for a good fit), rather than fitting to a theoretical equation, which would inevitably be based on assumptions that are not necessarily correct for all genotypes. The rate of recovery was characterized by the calculated (based on curve fitting) time necessary for half recovery (*t*_half_), as described (Lyubarsky and Pugh, 1996; Pepperberg et al., 1997; Song et al., 2009, 2011; Cleghorn et al., 2011).

### Statistical Analysis

All data presented here were obtained on three or more mice. To reduce variability of genetic background, the comparisons between lines expressing mutant rhodopsins with and without enhanced arrestin-1-3A were performed on littermates. The data were analyzed using StatView software (SAS Institute, Cary, NC, United States). The histological data were first analyzed by two-way repeated measure ANOVA with Genotype as the main factor and Retinal Subdivision as a repeated measure factor. One-way ANOVA was used to examine the effect of Genotype in each retinal subdivision separately. The intensities of a- and b-waves in ERG were analyzed by two-way repeated measure ANOVA with Genotype as between group and Light Intensity as within group factor. The *t*_half_ data were analyzed by one-way ANOVA with Genotype as the main factor. Means were compared using Bonferroni *post hoc* test with correction for multiple comparisons. In all cases, *p* < 0.05 was considered significant.

## Results and Discussion

We compared retinal histology and the function of rod photoreceptors in WT mice and mice transgenically expressing on the rhodopsin knockout background mutant mouse rhodopsin lacking all six phosphorylation sites in its C-terminus (CSM), as well as rhodopsin that has only one (Ser-338)(1P) or two (Ser-334 and Ser-338)(2P) phosphorylation sites (Mendez et al., 2000a). Light-induced signaling of these mutant forms of rhodopsin was found to be deactivated much slower than that of WT mouse rhodopsin carrying six phosphorylation sites in the presence of normal complement of WT arrestin-1 (Mendez et al., 2000a).

**FIGURE 1:**
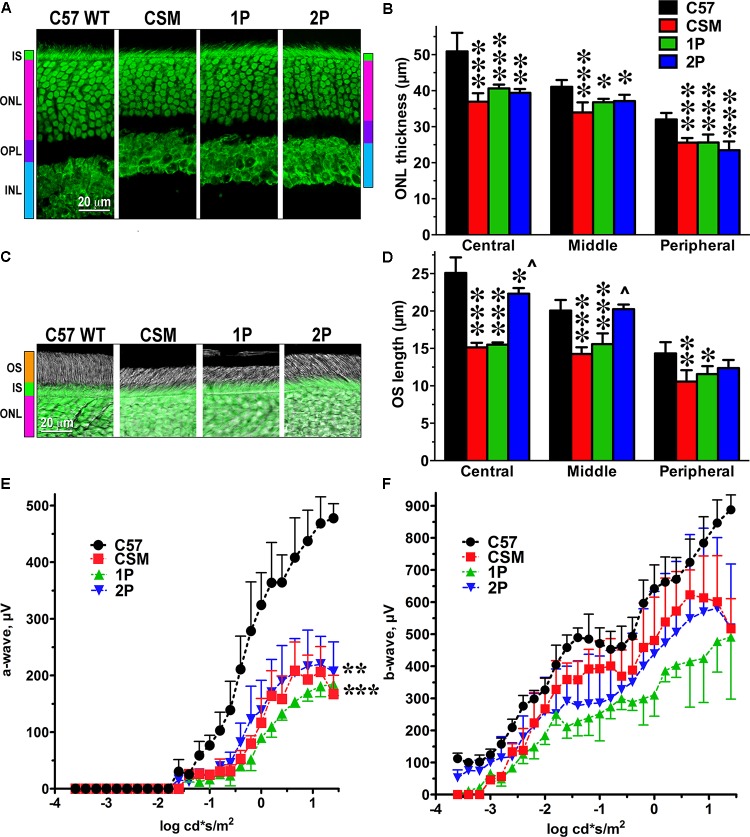
The effect of reduced number of rhodopsin phosphorylation sites on photoreceptor morphology and ERG parameters. **(A)** Confocal green fluorescent Nissl images of the ONL in central retina sections of 5–6 weeks old mice of indicated genotypes. The positions of the outer segments (OS), inner segments (IS), outer nuclear layer (ONL), outer plexiform layer (OPL), and inner nuclear layer (INL) are shown on the left. **(B)** The thickness of the ONL (reflecting the number of rod photoreceptors) measured in the Central, Middle, and Peripheral retina were the average of inferior and superior retinal hemispheres. Means ± SE from at least three animals per genotype are shown. The comparison of the thickness of the ONL separately for each retinal subdivision by one-way ANOVA with Genotype as main factor revealed significant effect of Genotype for Central, Middle, and Peripheral retina. ^∗^*p* < 0.05; ^∗∗^*p* < 0.01; ^∗∗∗^*p* < 0.001, as compared to WT C57 mice according to Bonferroni *post hoc* comparison. **(C)** Combined DIC and green fluorescent Nissl images of the retina sections of 5–6 weeks old mice of indicated genotypes, enlarged to show OS more clearly. The positions of outer segments (OS), inner segments (IS), and outer nuclear layer (ONL) are shown on the left. **(D)** The length of the OS measured in the Central, Middle, and Peripheral retina were the average of inferior and superior retinal hemispheres. Means ± SE from three animals per genotype are shown. The length of OS was compared separately for each retinal subdivision by one-way ANOVA with Genotype as main factor, followed by Bonferroni *post hoc* test. The effect of Genotype was significant in all retinal subdivisions (*p* < 0.0001). ^∗^*p* < 0.05; ^∗∗^*p* < 0.01; ^∗∗∗^*p* < 0.001, as compared to WT; ^∧^*p* < 0.001 to CSM and 2P. **(E)** Amplitude of the a-wave (reflecting direct response of photoreceptors to light) was lower than in WT in all lines with limited number of phosphorylation sites (two-way ANOVA with Genotype as the main factor and Light Intensity as the repeated measure factor; the Genotype effect was significant: F(3,112) = 20.73; *p* = 0.0004. The Light Intensity effect was also significant (*p* < 0.0001) and so was Genotype-Light Intensity interaction [F(28,112) = 13.3; *p* < 0.0001]. ^∗∗^*p* < 0.01; ^∗∗∗^*p* < 0.001 to WT across light intensities according to *post hoc* Bonferroni test. Means ± SE from three animals per genotype are shown. **(F)** The differences among genotypes in the amplitude of the b-wave, which reflects the response of the bipolar cells driven by both rods and cones, did not reach statistical significance [F(3,192) = 3.23; *p* = 0.082], but Genotype-Light Intensity interaction was significant [F(48,192) = 1.9; *p* = 0.0003]. Means ± SD of the data from three mice of each genotype are shown. Means ± SE from three animals per genotype are shown. The data from WT mice were published earlier (Song et al., 2009) and are used here for comparison.

We characterized retinal histology of these lines measuring the thickness of the ONL, which reflects the number of rod photoreceptors (**Figures 1A,B**), and the length of the outer segments, which reflects the rod health (**Figures 1C,D**). We found that in CSM, 1P, and 2P lines the ONL was thinner than in WT mice (C57) (**Figure 1A**). Although only in one case the reduction of the ONL thickness slightly exceeded 25% (in the central retina of CSM mice -27.4%), the loss of photoreceptors in other genotypes/retinal subdivisions was statistically highly significant (**Figure 1B**). The reduction of the outer segment length in CSM and 1P lines tended to be more dramatic (up to 40%), particularly in the central and middle retina (**Figures 1C,D**). Interestingly, 2P line demonstrated much longer outer segments that, except in the central retina, were not significantly shorter than the OS of WT C57 mice (**Figures 1C,D**).

Electrophysiologically, the lines expressing phosphorylation-deficient rhodopsin were only characterized using single-cell recordings (Mendez et al., 2000a). As we were primarily interested in the therapeutic potential of our approach, we aimed to evaluate the function of the whole retina, rather than individual photoreceptors. Therefore, we used ERG that reports field potential reflecting the light response of the entire retina. We found that in all three lines with insufficient rhodopsin phosphorylation the amplitude of the a-wave was significantly reduced, as compared to WT mice (**Figure 1E**). The reduction of the a-wave amplitude (**Figure 1E**) appeared to be more significant that the reduction of the length of the outer segments in these lines (**Figures 1C,D**). Conceivably, the surface density of the dark current in the remaining outer segments is lower than in WT, although we did not test this possibility. In case of b-wave, the differences did not reach statistical significance, but the Genotype x Light Intensity interaction was significant [F(48,192) = 1.9; *p* = 0.0003), indicating the difference in the shape of the curves (**Figure 1F**). As could be expected, the effect on the a-wave, which reflects direct rod response to light in the mouse retina, where cones constitute only 2–3% of the total photoreceptor complement (Nikonov et al., 2008), was greater than on the b-wave, which reflects the response of the bipolar cells that is driven by both rods and cones (**Figures 1E,F**). As far as a-wave is concerned, the effect of impaired rhodopsin phosphorylation appeared greater at lower light levels (below -0.5 log cd^∗^s/m^2^) (**Figure 1E**), where the contribution of presumably unaffected cones is negligible. The responses in the 2P line tended to be less affected than in the 1P line (**Figure 1E**).

**FIGURE 2:**
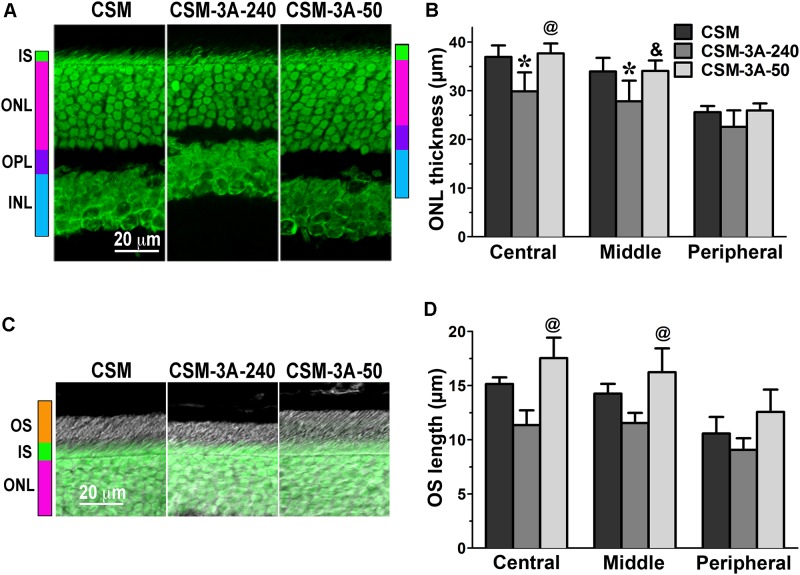
The effect of enhanced arrestin-1-3A on photoreceptors expressing rhodopsin with zero phosphorylation sites. **(A)** Confocal green fluorescent Nissl images of the ONL in central retina sections of 5–6 weeks old mice expressing rhodopsin without phosphorylation sites in the absence (CSM) or presence of low (CSM-3A-50) and high (3A-240) levels of arrestin-1-3A mutant. The positions of the outer segments (OS), inner segments (IS), outer nuclear layer (ONL), outer plexiform layer (OPL) and inner nuclear layer (INL) are shown on the left. **(B)** The thickness of the ONL (reflecting the number of rod photoreceptors) measured in the Central, Middle, and Peripheral retina were the average of inferior and superior retinal hemispheres. Means ± SE from three animals per genotype are shown. The comparison of the thickness of the ONL separately for each retinal subdivision by one-way ANOVA with Genotype as main factor revealed significant effect of Genotype for Central (*p* = 0.0058), Middle (*p* = 0.011), and Peripheral retina (*p* = 0.0489). ^∗^*p* < 0.05; ^∗∗^*p* < 0.01; ^∗∗∗^*p* < 0.001, as compared to CSM mice; ^&^*p* < 0.05, ^@^ *p* < 0.01 as compared to the 3A-240 line by Bonferroni *post hoc* comparison. **(C)** Combined DIC and green fluorescent Nissl images of the retina sections of 5–6 weeks old mice of indicated genotypes, enlarged to show OS more clearly. The positions of the outer segments (OS), inner segments (IS), and outer nuclear layer (ONL) are shown on the left. **(D)** The length of the OS measured in the Central, Middle, and Peripheral retina were the average of inferior and superior retinal hemispheres. Means ± SE from three animals per genotype are shown. The length of OS was compared separately for each retinal subdivision by one-way ANOVA with Genotype as main factor. The effect of Genotype was significant in the Central (*p* = 0.0047), Middle (*p* = 0.0039), but not Peripheral retina (*p* = 0.0866). ^@^ *p* < 0.01 as compared to the 3A-240 line by Bonferroni *post hoc* comparison.

**FIGURE 3:**
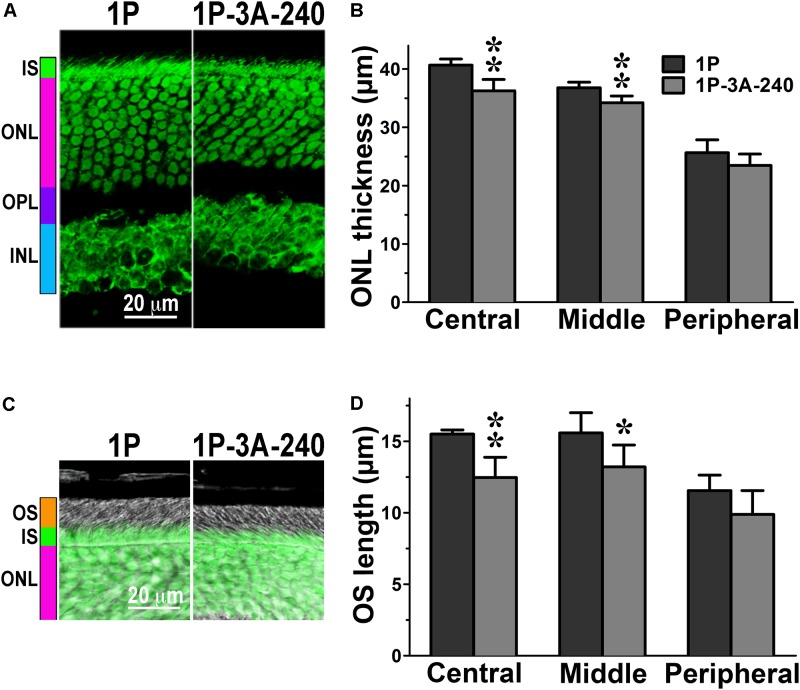
The effect of enhanced arrestin-1-3A on photoreceptors expressing mutant rhodopsin with one phosphorylation site. **(A)** Confocal green fluorescent Nissl images of the ONL in central retina sections of 5–6 weeks old mice expressing rhodopsin with one phosphorylation site (1P) in the absence or presence of high (1P-3A-240) levels of arrestin-1-3A mutant. The positions of the outer segments (OS), inner segments (IS), outer nuclear layer (ONL), outer plexiform layer (OPL) and inner nuclear layer (INL) are shown on the left. **(B)** The thickness of the ONL (reflecting the number of rod photoreceptors) measured in the Central, Middle, and Peripheral retina were the average of inferior and superior retinal hemispheres. Means ± SE from three animals per genotype are shown. The comparison of the thickness of the ONL separately for each retinal subdivision by one-way ANOVA with Genotype as main factor revealed significant effect of Genotype for the Central (*p* = 0.0022) and Middle (*p* = 0.005), but not Peripheral retina (*p* = 0.1393). ^∗^*p* < 0.05; ^∗∗^*p* < 0.01 as compared to 1P mice. **(C)** Combined DIC and green fluorescent Nissl images of the retina sections of 5–6 weeks old mice of indicated genotypes, enlarged to show OS more clearly. The positions of the outer segments (OS), inner segments (IS), and outer nuclear layer (ONL) are shown on the left. **(D)** The length of the OS measured in the Central, Middle, and Peripheral retina were the average of inferior and superior retinal hemispheres. Means ± SE from three animals per genotype are shown. The length of OS was compared separately for each retinal subdivision by two-tailed Student’s test The difference between the groups was significant in the Central (*p* = 0.0016) and Middle (0.0346) retinal subdivisions, but not in the Peripheral retina (*p* = 0.0968). ^∗^*p* < 0.05; ^∗∗^*p* < 0.01, as compared to 1P mice.

**FIGURE 4:**
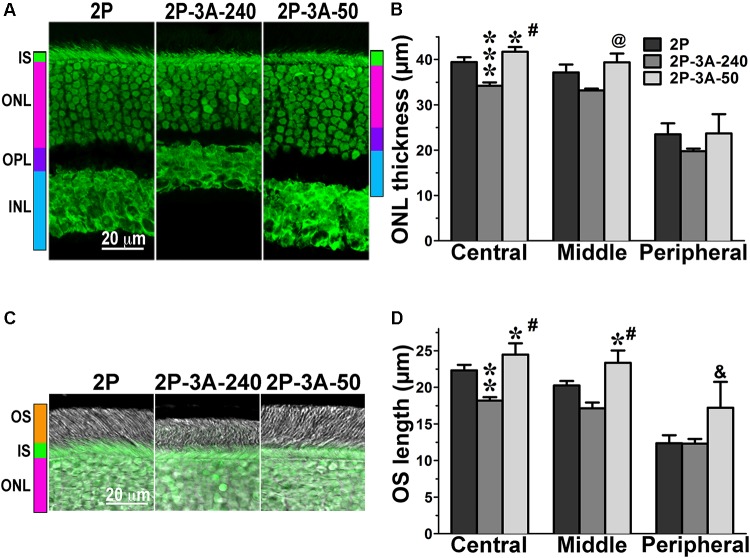
The effect of enhanced arrestin-1-3A on photoreceptors expressing mutant rhodopsin with two phosphorylation sites. **(A)** Confocal green fluorescent Nissl images of the ONL in central retina sections of 5–6 weeks old mice expressing rhodopsin with two phosphorylation sites (2P) in the absence or presence of low (2P-3A-50) and high (2P-3A-240) levels of arrestin-1-3A mutant. The positions of outer segments (OS), inner segments (IS), outer nuclear layer (ONL), outer plexiform layer (OPL) and inner nuclear layer (INL) are shown on the left. **(B)** The thickness of the ONL (reflecting the number of rod photoreceptors) measured in the Central, Middle, and Peripheral retina were the average of inferior and superior retinal hemispheres. Means ± SE from three animals per genotype are shown. The comparison of the thickness of the ONL separately for each retinal subdivision by one-way ANOVA with Genotype as main factor revealed significant effect of Genotype for the Central (*p* < 0.0001) and Middle (*p* = 0.0075), but not Peripheral retina (*p* = 0.3997). ^∗^*p* < 0.05; ^∗∗∗^*p* < 0.001, as compared to 2P mice; ^#^*p* < 0.001; ^@^ *p* < 0.01 to the 3A-240 line by Bonferroni *post hoc* comparison. **(C)** Combined DIC and green fluorescent Nissl images of the retina sections of 5–6 weeks old mice of indicated genotypes, enlarged to show OS more clearly. The positions of the outer segments (OS), inner segments (IS), and outer nuclear layer (ONL) are shown on the left. **(D)** The length of the OS measured in the Central, Middle, and Peripheral retina were the average of inferior and superior retinal hemispheres. Means ± SE from three animals per genotype are shown. The length of OS was compared separately for each retinal subdivision by one-way ANOVA with Genotype as main factor, followed by Bonferroni *post hoc* test. The effect of Genotype was significant in all retinal subdivisions: the Central (*p* = 0.0003), Middle (0.0006), and Peripheral (0.027) retina. ^∗^*p* < 0.05; ^∗∗^*p* < 0.01, as compared to 2P animals; ^#^*p* < 0.001; ^&^*p* < 0.05 to the 3A-240 line by Bonferroni *post hoc* comparison.

**FIGURE 5:**
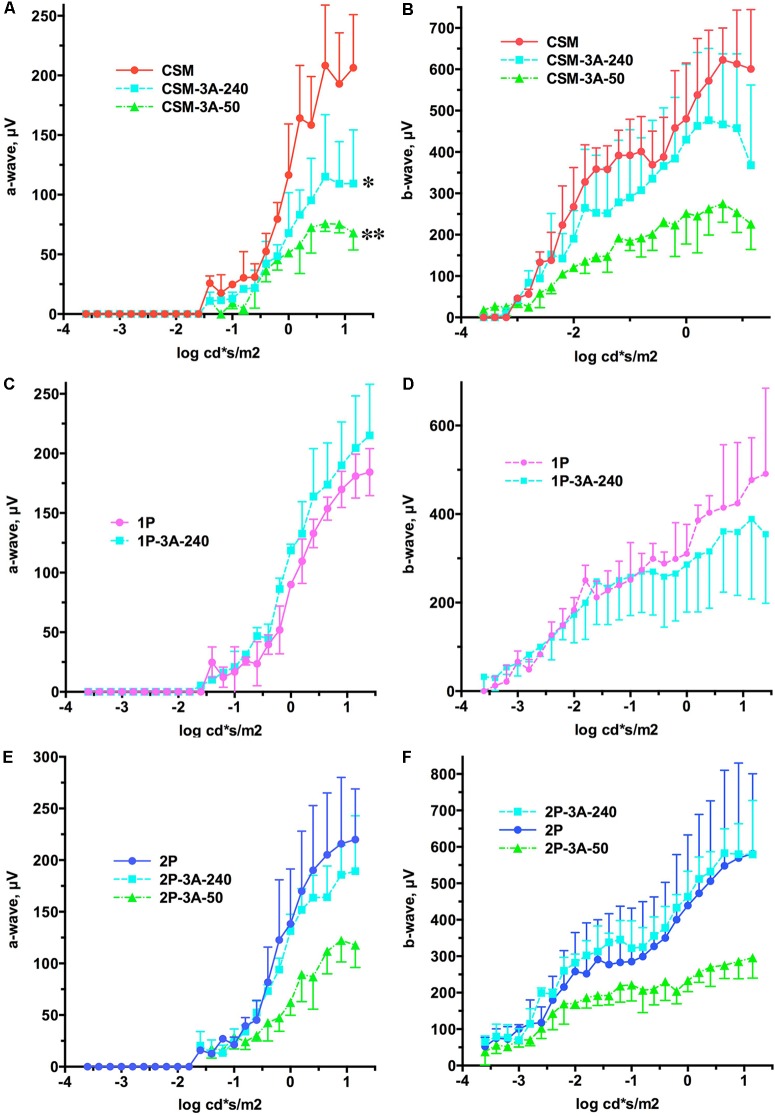
The effect of the expression of arrestin-1-3A on a- and b-wave of CSM, 1P, and 2P mice. **(A)** CSM a-wave. The effect of Genotype was significant [F(2,72) = 15.74; *p* = 0.0041]. Unexpectedly, the amplitude of the a-wave across light intensities was lower in CSM-3A-240 (^∗^*p* < 0.05) and CSM-3A-50 (^∗∗^*p* < 0.01) lines. **(B)** CSM b-wave. The effect of Genotype was not significant [F(2,138) = 3.76; *p* = 0.0874], but the interaction between Genotype and light intensity was [F(46,138) = 4.1; *p* < 0.0001], suggesting that the shapes of the curves are different. **(C)** 1P a-wave. Neither Genotype [F(1,56) = 2.85; *p* = 0.167], nor Genotype × light intensity interaction [F(28,56) = 1.01; *p* = 0.456] were significant. **(D)** 1P b-wave. Neither Genotype [F(1,96) = 0.152; *p* = 0.72], nor Genotype × light intensity interaction [F(28,96) = 0.958; *p* = 0.527] were significant. **(E)** 2P a-wave. The effect of Genotype was not significant [F(2,78) = 4.89; *p* = 0.055], but the interaction between Genotype and light intensity was [F(26,78) = 3.1; *p* < 0.0001]. **(F)** 2P b-wave. The effect of Genotype was not significant [F(2,138) = 2.81; *p* = 0.138], but the interaction between Genotype and light intensity was [F(46,138) = 2.6; *p* < 0.0001]. In all panels the color of lines expressing arrestin-1-3A indicates its level of expression (240% of WT, light blue; 50% of WT, green). Means ± SD of the data from three mice of each genotype are shown in all cases.

Having detected a clear phenotype in CSM, 1P, and 2P lines that express normal complement of endogenous WT arrestin-1 (**Figure 1**), next we tested whether co-expression of enhanced phosphorylation-independent arrestin-1-3A mutant can rescue it, as it partially rescued the deficits in rhodopsin kinase knockout animals (Song et al., 2009). To this end, we bred the mice transgenically expressing this mutant at ∼50% (3A-50) and 240% (3A-240) of WT arrestin-1 level (Song et al., 2009, 2013) with the lines expressing phosphorylation-deficient rhodopsin mutants on rhodopsin knockout background. We analyzed retinal histology of these lines (**Figures 2**–**4**) and measured the amplitudes of a- and b-wave in these animals (**Figure 5**).

In agreement with our previous findings on the background of WT rhodopsin (Song et al., 2013), on the CSM background we detected small, but significant reductions of the ONL thickness in animals expressing high level of 3A mutant (3A-240) in the central and middle retina, as compared to the base CSM line (**Figures 2A,B**). A lower expression of the same mutant in the CSM-3A-50 line did not affect the ONL thickness, as the CSM-3A-50 line did not differ from the base CSM line but was significantly different from the CSM-3A-240 line (**Figures 2A,B**). Interestingly, the OS were also shorter in CSM-3A-240 line, as compared to CSM-3A-50 animals (but not the base CSM line) (**Figures 2C,D**). Again, this effect was observed in the central and middle retina, whereas no significant differences in ONL thickness or OS length were detected in the peripheral retina (**Figure 2**). Similarly, in the central and middle retina high expression of enhanced 3A mutant (3A-240) reduced the thickness of the ONL (**Figures 3A,B**) and OS length (**Figures 3C,D**) on the 1P background. The effects of enhanced arrestin-1-3A expression on the retina of 2P mice **Figure 4**), that had higher numbers of healthier rod photoreceptors than CSM or 1P animals (**Figure 1**), were more complex. The high expression (in 3A-240 lines) reduced ONL thickness and OS length, but only in the central retina, whereas the differences between the 2P and 2P-3A-240 lines in the middle and peripheral retina did not reach statistical significance (**Figures 4A–D**). Importantly, on the 2P background lower expression of 3A mutant (3A-50 lines) actually increased ONL thickness in the central retina, as well as OS length in the central and middle retina (**Figures 4A–D**). Thus, a high expression (3A-240 lines) had a generally detrimental effect of the retinal morphology, whereas lower expression of the same enhanced mutant (3A-50 lines) appeared harmless or even beneficial promoting the photoreceptor survival and preservation of their outer segments.

We previously showed that a lower expression level of enhanced arrestin-1-3A mutant (3A-50 lines) in mice expressing normal rhodopsin but lacking rhodopsin kinase and endogenous arrestin-1 increased the amplitudes of both a- and b-waves, whereas high expression (3A-240) did not (Song et al., 2009). Therefore, here we compared the effects of these two levels of enhanced mutant expression in animals with deficits in rhodopsin phosphorylation that have endogenous WT arrestin-1, like human patients with mutations in rhodopsin (Apfelstedt-Sylla et al., 1993). We found that co-expression of the arrestin-1-3A mutant did not increase the amplitude of a- and b-wave in any of the lines (**Figure 5**). In fact, both levels of arrestin-1-3A in CSM-3A-50 and CSM-3A-240 lines actually reduced the response to light, as compared to CSM mice (**Figures 5A,B**). Higher expression of enhanced arrestin-1 mutant in 1P-3A-240 and 2P-3A-240 appeared harmless, although it did not rescue WT a- and b-wave amplitude, as would be expected in case of full compensation (**Figures 5A–F**). Thus, opposing effects of the two levels of enhanced arrestin-1-3A expression on retinal histology (**Figures 2**–**4**) did not translate into corresponding changes in the amplitude of a- and b-wave in animals with defective rhodopsin phosphorylation (**Figure 5**). Enhanced arrestin-1-3A mutant did not increase the amplitude of the a-wave to WT levels, suggesting that it did not prevent rod damage by phosphorylation-deficient rhodopsin (compare **Figures 1**, **5**).

**FIGURE 6:**
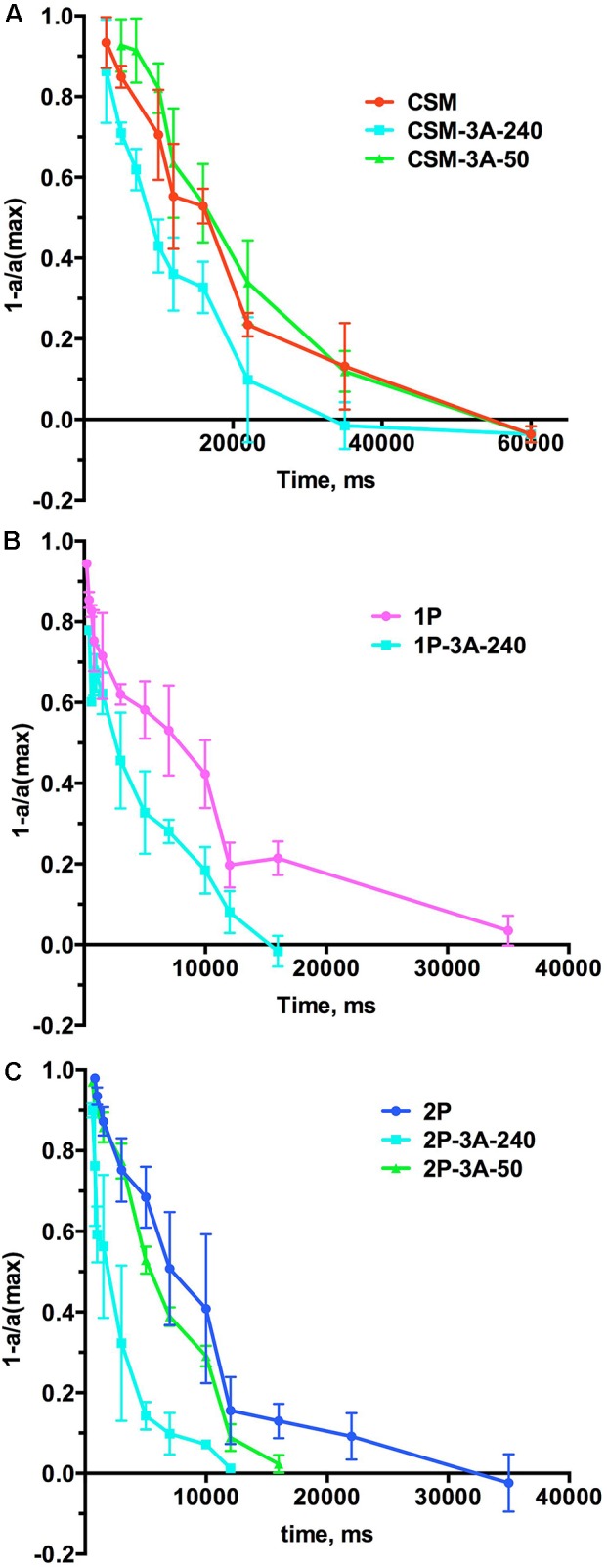
The effect of the expression of arrestin-1-3A on photoresponse recovery. **(A)** The comparison of CSM line with CSM-3A-240 and CSM-3A-50. **(B)** The comparison of 1P line with 1P-3A-240. **(C)**. The comparison of 2P line with 2P-3A-240 and 2P-3A-50. Means ± SD of the data from three mice of each genotype are shown. In all panels the color of lines expressing arrestin-1-3A indicates its level of expression (240% of WT, light blue; 50% of WT, green). See **Figure 7** for the quantification of the times of half recovery.

As arrestin-1 plays key role in photoresponse recovery (Gross and Burns, 2010; Gurevich et al., 2011), next we compared the recovery kinetics in the CSM, 1P, and 2P lines and the lines co-expressing these rhodopsin mutants with enhanced phosphorylation-independent arrestin-1-3A using double-flash ERG, where the first flash desensitizes rods, and the response to the second flash, delivered at different times after the first, is used to gauge the level of photoreceptor recovery (**Figure 6**). The results were plotted as one minus the ratio of measured (a) to maximum (a_max_) response as a function of time between flashes, so that in these graphs 1 represents total lack of response, whereas 0 represents full recovery (**Figure 6**). In these experiments, we detected clear effect of the high expression of arrestin-1-3A mutant: the recovery was much faster in CSM-3A-240, 1P-3A-240, and 2P-3A-240 lines than in CSM, 1P, or 2P animals, respectively (**Figure 6**), reflecting an enhanced ability of arrestin 3A mutant in deactivating rhodopsin with little or no phosphorylation. Interestingly, lower expression of the mutant was ineffective: the rate of recovery in CSM-3A-50 and 2P-3A-50 lines was indistinguishable from that in CSM and 2P lines, respectively (**Figure 6**).

**FIGURE 7:**
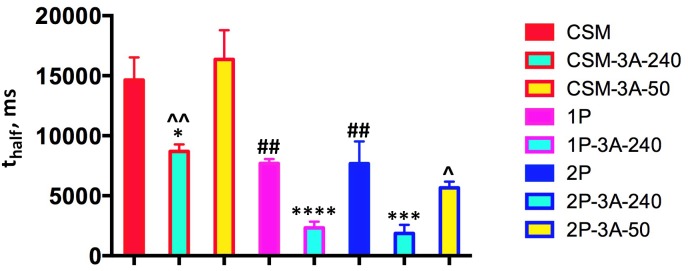
High expression of enhanced arrestin-1-3A mutant facilitates recovery of rods expressing phosphorylation-deficient rhodopsin. The times of half-recovery (*t*_half_) were, as follows: CSM, 14.65 ± 1.87 s; CSM-3A-240, 8.68 ± 0.58; CSM-3A-50, 16.36 ± 2.43; 1P, 7.70 ± 0.36; 1P-3A-240, 2.32 ± 0.53; 2P, 7.68 ± 1.85; 2P-3A-240, 1.86 ± 0.72; 2P-3A-50, 5.66 ± 0.52. The data were analyzed by one-way ANOVA in groups with Genotype as the main factor. The effect of Genotype in CMS-1P-2P group was significant [F(2,6) = 20.7; *p* = 0.002]. According to Bonferroni *post hoc* test, *t*_half_ in both 1P and 2P was shorter than in CMS (^##^*p* < 0.01). Within CMS group (including CMS, CMS-3A-240 and CMS-3A-50), the effect of Genotype was also significant [F(2,6) = 15, *p* = 0.0046], and according to Bonferroni test CMS-3A-240 was different from both CMS (^∗^*p* = 0.05) and CMS-3A-50 (ˆˆ*p* = 0.01). In 1P group *t*_half_ of 1P-3A-240 was significantly shorter that of parental 1P line (^∗∗∗∗^*p* = 0.0001). In the 2P group the effect of Genotype was also significant [F(2,6) = 18.7; *p* = 0.0026], with *t*_half_ of 2P-3A-240 line was significantly shorter than in 2P (^∗∗∗^*p* = 0.001) and 2P-3A-50 line (ˆ*p* = 0.05). The color of the outline corresponds to parental line (see legend to **Figure 1**), whereas the fill color indicates the level of arrestin-1-3A expression (240% of WT, light blue; 50% of WT, yellow). Means ± SD of the data from three mice of each genotype are shown.

The rate of recovery is usually expressed as *t*_half_, which represents the time necessary for half recovery (i.e., the time at which the response reaches half of its maximum value). Unbiased fitting of the recovery curves (**Figure 6**) by polynomial non-linear regression (that does not make any assumptions regarding the shape of the curve) yielded for the CSM line, where rhodopsin has no phosphorylation sites, a *t*_half_ value of 14.65 ± 1.87 s (**Figure 7**). This is statistically indistinguishable from the *t*_half_ in rhodopsin kinase knockout animals (18.67 ± 3.28 s) we measured earlier (Song et al., 2009), which lack rhodopsin phosphorylation for a different reason. Measured *t*_half_ of both 1P and 2P lines were significantly shorter (7.70 ± 0.36 and 7.68 ± 1.85 s, respectively) than in the CSM line (**Figure 7**), in agreement with faster recovery suggested by published single cell data (Mendez et al., 2000a). High expression of enhanced arrestin-1-3A mutant significantly facilitated the recovery in all cases, almost two-fold in the CSM and more than 3-fold in the 1P and 2P animals (**Figure 7**). In contrast, lower expression level in the CSM-3A-50 and 2P-3A-50 lines did not accelerate photoresponse recovery: the rates remained essentially the same as in the CSM and 2P lines, respectively (**Figure 7**). Where tested (the CSM and 2P backgrounds), the recovery in mice expressing high level (240% of WT) of enhanced arrestin-1-3A was significantly faster than in animals expressing the same mutant at 50% of WT level (**Figure 7**). In the previous study (Song et al., 2009), we have demonstrated that the phosphorylation-independent arrestin-1-3A mutant compensated for the defects in rhodopsin phosphorylation caused by the lack of rhodopsin kinase (on the arrestin knockout background). The compensation was partial reducing *t*_half_ from ∼19 s to ∼10 s and ∼6 s in lines expressing 3A mutant at high (240% of WT) and low (50% of WT) levels, respectively. Thus, in the absence of endogenous WT arrestin-1 the mutant expressed at 50% of WT level worked better than at 240% (Song et al., 2009), which was consistent with rapid light-independent retinal degeneration caused by the high mutant expression in the absence of WT arrestin-1 (Song et al., 2013). In this study, the defect in rhodopsin phosphorylation was caused by the absence of all (CSM) or all but 1 (1P) or 2 (2P) phosphorylation sites in mice expressing normal level of WT arrestin-1. We found that the phosphorylation-independent arrestin-1-3A mutant was also able to compensate for this defect, but only when expressed at higher level (240% of WT), whereas lower level (50% of WT) proved ineffective (**Figures 6**, **7**). The fastest recovery in the “compensated” retina was in the 2P-3A-240 line, where *t*_half_ was 1.86 ± 0.72 s. While this sounds impressive when compared to the original *t*_half_ in the 2P line, which was 7.68 ± 1.85 s, it is still much longer than 0.38 ± 0.03 s in WT mice under similar conditions (Song et al., 2009).

Interestingly, high expression of the phosphorylation-independent arrestin-1-3A mutant caused retinal degeneration in both conditions. We have previously observed rapid age-dependent but light-independent loss of photoreceptors, their outer segments and their terminal fields (outer plexiform layer) in mice expressing the arrestin-1-3A mutant at 240% of WT on the arrestin-1 knockout background (Song et al., 2009, 2013). We also found that co-expression of WT arrestin-1 offers protection, albeit incomplete, against the arrestin-1-3A-induced photoreceptor degeneration (Song et al., 2013). In this study, high expression of the 1-3A mutant also lead to the retinal degeneration, but the degree was limited, presumably due to the presence of WT arrestin-1, in agreement with our previous results (Song et al., 2013). Previously we did not observe that lower expression of the 3-A mutant on the arrestin-1 knockout background caused any deleterious effect until mice were about 32 weeks of age (Song et al., 2013). Here we find that co-expression of the 3A mutant at a modest level with WT arrestin-1 not only does not impair but actually improves the retinal morphology, at least, in some rhodopsin phosphorylation-deficient lines. This effect could again be due to the protective action of WT arrestin-1 more evident with the lower concentration of the mutant. To explain our data, we proposed that monomeric arrestin is toxic to rod photoreceptors and that oligomerization of arrestin-1 is a neuroprotective mechanism (Song et al., 2013). Since 3A arrestin-1 is partially deficient in oligomerization, at high concentrations it causes rod apoptosis and retinal degeneration. WT arrestin-1 offers protection against the 3A mutant toxicity because of its ability to take oligomerization-deficient mutants into mixed oligomers. A later study suggested that the ability of arrestin-1 to bind clathrin adaptor AP2 via its C-terminus causes photoreceptor death (Moaven et al., 2013). Indeed, in the arrestin-1-rhodopsin complex, the arrestin C-terminus is detached and free to interact with other partners (Kang et al., 2015; Zhou et al., 2017). Based on the crystal structures of arrestin-1 (Hirsch et al., 1999), the C-terminus must be detached and fully accessible in the 3A mutant, and could, therefore, be engaged in interaction with AP2. This likely explains the concentration-dependent adverse effects of the 3A mutant that manifest themselves regardless of light exposure (Song et al., 2013). It is also likely that in case of the 3A mutant both mechanisms contribute to its toxicity.

The arrestin-1 self-association appears to be the most parsimonious explanation of observed discrepancy between the effect of the expression level of enhanced arrestin-1-3A mutant in lines that do or do not express endogenous WT arrestin-1. The common feature of bovine, mouse, and human arrestin-1 is the formation of dimers and tetramers (Hanson et al., 2007a,b, 2008; Kim et al., 2011). Curiously, arrestin-1 self-association was described even before the arrestin function became clear, when this protein was still called S-antigen for its role in uveitis (Wacker et al., 1977). In fact, at millimolar concentrations of arrestin-1 in rod photoreceptors (Strissel et al., 2006; Hanson et al., 2007a; Song et al., 2011) the great majority of it exists in oligomeric forms (Kim et al., 2011), whereas only monomer was shown to bind rhodopsin (Hanson et al., 2007b). Although 3A mutation *per se* reduces arrestin-1 propensity to self-associate, it does not abolish it (Vishnivetskiy et al., 2013). Apparently, when the overall concentration of the enhanced mutant is lower than that of the endogenous arrestin-1, most of it is “sucked into” mixed oligomers with the WT, and therefore cannot quench the signaling of unphosphorylated or insufficiently phosphorylated rhodopsin. In contrast, in the lines expressing the mutant at 240% of WT, the majority of arrestin-1 is the enhanced mutant, which produces sufficient concentration of mutant monomer to dampen the signaling of rhodopsin lacking sufficient number of phosphorylation sites. We earlier found that as low as 5% of WT level of arrestin-1 effectively quenches rhodopsin signaling after the flashes of moderate intensity used here (Cleghorn et al., 2011). This explanation is tentatively supported by the fact that a low level of the 3A mutant was sufficient to preserve or even slightly improve the retinal morphology, although it offered no functional benefits in terms of the reduction in the recovery time. In contrast, at 240% the 3A mutant significantly improved the recovery time in spite of its detrimental effect on the photoreceptor survival and health.

Human patients with mutations in rhodopsin that eliminate or limit the number of phosphorylation sites in the rhodopsin C-terminus (Apfelstedt-Sylla et al., 1993) express normal levels of WT arrestin-1. High expression of pre-activated mutant is harmful for rods even in the presence of WT arrestin-1 (Song et al., 2013), whereas low levels do not compensate in the presence of WT arrestin-1, presumably, because it is tied up in the mixed oligomers (**Figures 6**, **7**). Thus, for effective gene therapy enhanced arrestin-1 must be rendered oligomerization-deficient. Double alanine substitution of interacting phenylalanines in bovine and mouse arrestin-1, based on the elucidated solution structure of arrestin-1 tetramer (Hanson et al., 2008) [which is quite different from that of the crystal tetramer (Granzin et al., 1998; Hirsch et al., 1999)] prevents arrestin-1 self-association (Kim et al., 2011). These mutations render enhanced arrestin-1 mutants oligomerization-deficient without significantly affecting their ability to bind unphosphorylated rhodopsin (Vishnivetskiy et al., 2013). Thus, only arrestin-1 mutants combining high affinity for the unphosphorylated rhodopsin with inability to self-associate have potential to serve as tools for compensational gene therapy in patients expressing rhodopsin that cannot be quenched by the normal mechanism. For safety, the AP2 binding site in these mutants should be deleted or disabled, and the expression levels should be kept relatively low, at ∼5–10% of WT.

To summarize, enhanced phosphorylation-independent arrestin-1-3A mutant partially compensates for various defect of rhodopsin phosphorylation *in vivo*: the absence of rhodopsin kinase (Song et al., 2009), as well as the absence (CSM) or insufficient number (1P and 2P) of phosphorylation sites in the rhodopsin itself (**Figures 6**, **7**). However, in contrast to arrestin-1 knockout lines (Song et al., 2009), in the presence of normal complement of WT arrestin-1 mimicking the situation in human patients, only high expression of the mutant is effective. As supra-physiological expression of arrestin-1-3A *per se* causes photoreceptor death via light-independent mechanism (Song et al., 2013), it appears that to serve as a therapeutic tool, enhanced arrestin-1 must be rendered oligomerization-deficient and lack the AP2 binding site.

## Author Contributions

SS, XS, and SV performed the experiments and analyzed the data. EG performed the statistical analysis. JC generated mouse strains expressing rhodopsin mutants. JC, EG, and VG designed the experiments and wrote the manuscript with input from other authors.

## Conflict of Interest Statement

The authors declare that the research was conducted in the absence of any commercial or financial relationships that could be construed as a potential conflict of interest.

## Footnotes

1 Different names of arrestin proteins are used in the field. We use the systematic names of arrestins, where the number after the dash indicates the order of cloning: arrestin-1 (historic names S-antigen, 48-kDa protein, visual or rod arrestin), arrestin-2 (β-arrestin or β-arrestin1), arrestin-3 (β-arrestin2 or hTHY-ARRX), and arrestin-4 (cone or X-arrestin).
